# Hexosamine biosynthesis and related pathways, protein N-glycosylation and O-GlcNAcylation: their interconnection and role in plants

**DOI:** 10.3389/fpls.2024.1349064

**Published:** 2024-03-06

**Authors:** Ya-Huei Chen, Wan-Hsing Cheng

**Affiliations:** Institute of Plant and Microbial Biology, Academia Sinica, Taipei, Taiwan

**Keywords:** N-acetylglucosamine, hexosamine biosynthesis pathway, salvage pathway, N-glycosylation, O-GlcNAcylation, abiotic stress

## Abstract

N-Acetylglucosamine (GlcNAc), a fundamental amino sugar moiety, is essential for protein glycosylation, glycolipid, GPI-anchor protein, and cell wall components. Uridine diphosphate-GlcNAc (UDP-GlcNAc), an active form of GlcNAc, is synthesized through the hexosamine biosynthesis pathway (HBP). Although HBP is highly conserved across organisms, the enzymes involved perform subtly distinct functions among microbes, mammals, and plants. A complete block of HBP normally causes lethality in any life form, reflecting the pivotal role of HBP in the normal growth and development of organisms. Although HBP is mainly composed of four biochemical reactions, HBP is exquisitely regulated to maintain the homeostasis of UDP-GlcNAc content. As HBP utilizes substrates including fructose-6-P, glutamine, acetyl-CoA, and UTP, endogenous nutrient/energy metabolites may be integrated to better suit internal growth and development, and external environmental stimuli. Although the genes encoding HBP enzymes are well characterized in microbes and mammals, they were less understood in higher plants in the past. As the HBP-related genes/enzymes have largely been characterized in higher plants in recent years, in this review we update the latest advances in the functions of the HBP-related genes in higher plants. In addition, HBP’s salvage pathway and GlcNAc-mediated two major co- or post-translational modifications, N-glycosylation and O-GlcNAcylation, are also included in this review. Further knowledge on the function of HBP and its product conjugates, and the mechanisms underlying their response to deleterious environments might provide an alternative strategy for agricultural biofortification in the future.

## 1 Introduction

**Figure 1:**
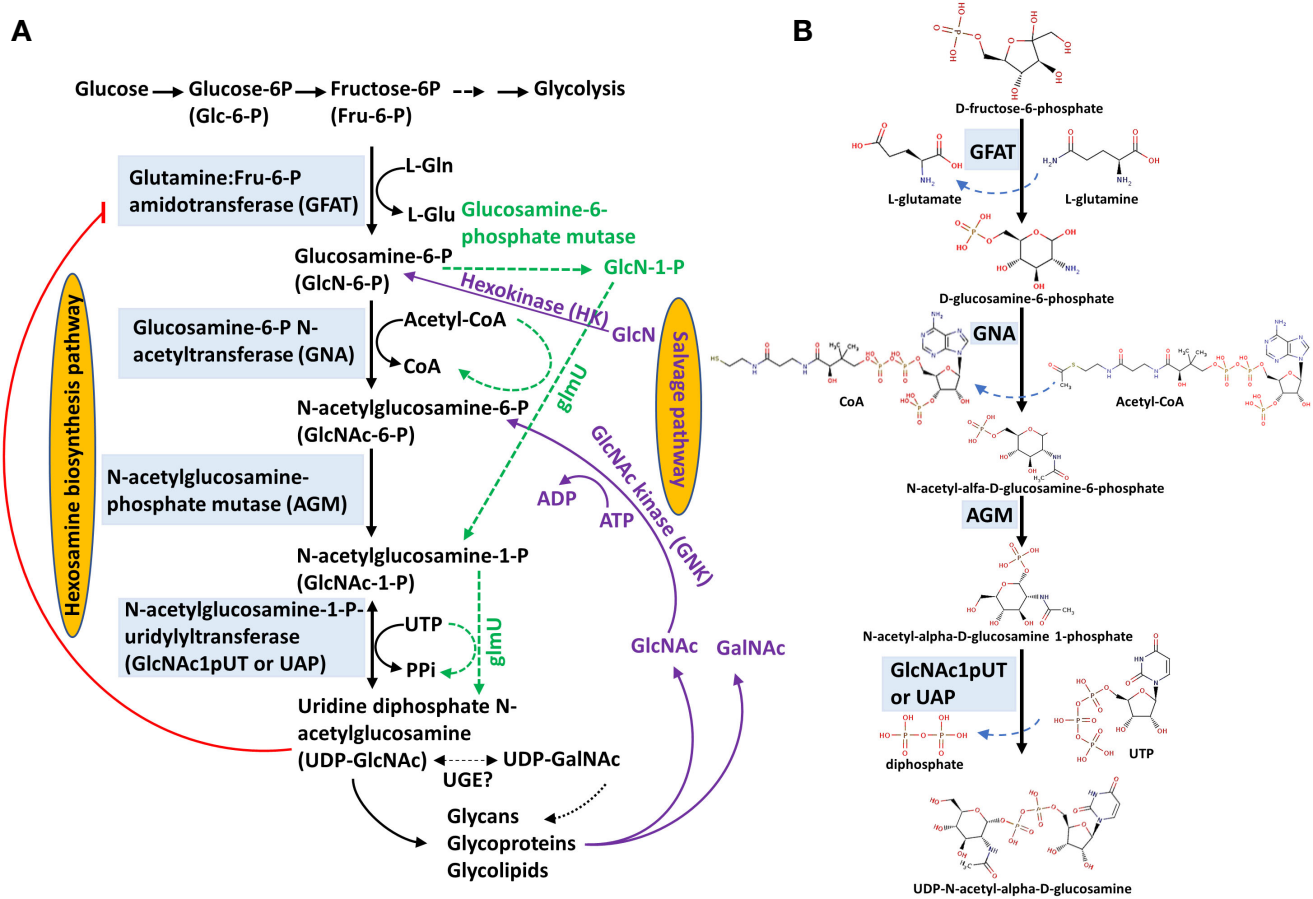
UDP-GlcNAc biosynthesis through hexosamine biosynthesis and salvage pathways. **(A)** Hexosamine biosynthesis and salvage pathways. The hexosamine biosynthesis pathway (HBP) is composed of four reactions catalyzed sequentially by glutamine:Fru-6-P amidotransferase (GFAT), glucosamine-6-P N-acetyltransferase (GNA), N-acetylglucosamine-phosphate mutase (AGM) and N-acetylglucosamine-1-P uridylyltransferase (GlcNAc1pUT or UAP) to synthesize uridine diphosphate N-acetylglucosamine (UDP-GlcNAc). UDP-GlcNAc is presumably interconverted to UDP-N-acetylgalactosamine (UDP-GalNAc) by an uncharacterized UDP-Glc-4-epimerase (UGE) in plants. In the salvage pathway (purple lines), GlcN is used and converted to GlcN-6-P catalyzed by a hexokinase (HK), followed by entering HBP to form UDP-GlcNAc. In addition, GlcNAc can be converted to GlcNAc-6-P catalyzed by an N-acetylglucosamine kinase (GNK); GlcNAc-6-P further enters the HBP to form UDP-GlcNAc. The green dashed lines represent HBP in prokaryotes. This HBP diagram is modified from Furo et al. (2015) and Chen et al. (2022). **(B)** Biochemical structures of HBP. These chemical structures are derived from the BRENDA database (https://www.brenda-enzymes.org/fulltext.php?overall=1).

Plants, as sessile organisms, frequently suffer from deleterious environmental stimuli. Many cellular metabolic processes, such as carbohydrates, amino acids, lipids, and energy metabolism, are influenced by different developmental stages and abiotic stresses (Van Zelm et al., 2020; Mansour and Hassan, 2022). In response to developmental changes and external challenges, plants have evolved sophisticated mechanisms to better suit plant growth and environmental changes by integrating their internal metabolic status and optimizing metabolic reprogramming. One of these metabolic processes is the so-called hexosamine biosynthesis pathway (HBP), which utilizes fructose-6-phosphate (Fru-6-P), glutamine, acetyl-coenzyme A (acetyl-CoA), and uridine triphosphate (UTP) as substrates to synthesize uridine diphosphate-N-acetylglucosamine (UDP-GlcNAc) (**Figure 1**). As the metabolic flux through HBP integrates glycolysis, amino acid, lipid, and nucleic acid pathways to maintain their balance and keep UDP-GlcNAc homeostasis, HBP may function as a metabolic integrator or hub for sensing nutrients (Buse, 2006; Chiaradonna et al., 2018) to link cellular nutrients/or energy signals and external cues. The HBP flux that generates UDP-GlcNAc is primarily regulated by the rate-limiting enzyme glutamine:fructose-6-phosphate amidotransferase (GFAT) activity and the obligatory substrate of O-linked GlcNAc transferase (OGT). The increased flux through HBP might be linked to insulin resistance, the vascular complications of diabetes, and cancer formation in mammals (Buse, 2006; Chiaradonna et al., 2018).

UDP-GlcNAc, the active form of GlcNAc, is a fundamental amino sugar moiety essential for the glycosylation of proteins and lipids (Ebert et al., 2018), glycosylphosphatidylinositol (GPI)-anchor proteins (Lalanne et al., 2004), a cell wall component of chitin in yeast, and an exoskeleton of arthropods (Maia, 1994; Kato et al., 2002; Arakane et al., 2011). UDP-GlcNAc is synthesized through an HBP that involves four consecutive reactions orderly catalyzed by a GFAT (Hassid et al., 1959; Durand et al., 2008), a glucosamine-6-P N-acetyltransferase (GNA) (Vetting et al., 2005; Wang et al., 2008a), an N-acetylglucosamine-phosphate mutase (AGM)/or phosphoacetylglucosamine mutase (Mio et al., 2000), and an N-acetylglucosmine-1-P-uridylyltransferase (GlcNAc1pUT, Yang et al., 2010) or a UDP-N-acetylglucosamine pyrophosphorylase (UAP; Wang et al., 2015, Wang et al., 2021). These processes are essential for cell growth and stress response and are conserved across organisms (Milewski et al., 2006). Dysfunction of HBP enzymes frequently causes severe phenotypes (Schimmelpfeng et al., 2006); moreover, a complete block of HBP normally results in lethality in yeast, mammals, and plants (Milewski et al., 2006; Chen et al., 2014; Pantaleon, 2015; Vu et al., 2019; Jia et al., 2023).

In the past, the function and regulation of HBP have been more intensively studied in microbes and mammals than in plants. The application of HBP products or intermediates through dietary treatment has been proposed as a therapy for human genetic disorders (Paneque et al., 2023). Elegant reviews have been recently reported on the function and regulation of HBP, which are primarily stressed in microbes (Wyllie et al., 2022) and eukaryotes of mammals (Paneque et al., 2023); however, an overview of HBP functions in plants is lacking. As research progress has greatly advanced in plant HBP study in recent years, in this review, we update our current knowledge of HBP function in plants and its stress responses. In addition, we provide an overview of the salvage pathway of HBP and the targets of UDP-GlcNAc in two major co- or post-translational modifications, N-linked glycosylation (N-glycosylation) and O-linked β-N-acetylglucosamine (O-GlcNAcylation). Further knowledge of HBP function and its response to abiotic stress may provide an alternative strategy to manipulate plant growth and tolerance to abiotic stress.

## 2 Hexosamine biosynthesis pathway enzymes

### 2.1 L-glutamine:D-fructose-6-phosphate amidotransferase

The first committed step of HBP is the transamination of D-fructose-6-phosphate from L-glutamine to form D-glucoamine-6-phosphate and L-glutamate, catalyzed by an L-glutamine:D-fructose-6-phosphate amidotransferase (GFAT; EC2.6.1.16) (**Figure 1**), also known as glucosamine-6-phosphate synthase (GlcN6P synthase). GFAT acts as the rate-limiting enzyme in the *de novo* HBP in fungi and animals (Olchowy et al., 2007; Walter et al., 2020; Paneque et al., 2023). Based on its origins in prokaryotes and lower or higher eukaryotes, the GFAT-encoded gene has been termed *Glms*, *GFA*, or *GFAT* (Durand et al., 2008). The function of GFAT is conserved among organisms, including microorganisms, mammals, and plants (Milewski et al., 2006). Yeast GFA1 activity is inhibited by UDP-GlcNAc and this inhibition is noncompetitive. In the pathogenic yeast *Candida albicans*, GFA activity increases during the yeast-to-mycelium morphological transformation, ensuring that UDP-GlcNAc production is increased when more amino sugars are needed in mycelium cells (Milewski et al., 1999, Milewski et al., 2006). GFA1 is the primary target molecule of methylmercury in *Saccharomyces cerevisiae* and yeast cells overexpressing *GFA1* confer resistance to methylmercury, an important environmental pollutant that causes neurological toxicity in mammals (Miura et al., 1999; Naganuma et al., 2000).

In plants, GFAT activity was first described by Hassid et al. (1959), and GFAT activity from mung bean *Phaseolus aureus* was partially purified and characterized (Vessal and Hassid, 1972). The Arabidopsis genome only contains a single copy of the *GFAT* gene (At3g24090), termed *GFAT1*, and its expression is primarily restricted to mature pollen grains (Wang et al., 2008b; Vu et al., 2019). Nevertheless, Arabidopsis *GFAT1* transcripts are also detectable in roots, flowers, and siliques by reverse transcription-quantitative polymerase chain reaction (RT-qPCR) (Jia et al., 2023). The loss-of-function *AtGFAT1* displays defects in a polar deposition of pectin and callose in the pollen wall, leading to inactivation of pollen activity; thus, the knockout mutant *Atgfat1-2* is lethal. In contrast, the knockdown mutant *Atgfat1* or *GFAT1* RNAi lines show reductions in glucosamine (GlcN) and UDP-GlcNAc levels in association with the reduced protein N-glycosylation but increased sensitivity of tunicamycin, an ER stress inducer agent. The RNAi lines also impair vegetative and reproductive development and display partial sterility. The abnormal phenotypes observed in *Atgfat1* can be largely rescued by the exogenous application of GlcN (Vu et al., 2019). It was reported that GlcN inhibits Arabidopsis hypocotyl elongation due to the induction of reactive oxygen species (ROS). Arabidopsis transgenic plants overexpress *E. coli glucosamine-6-phosphate deaminase* (*NagB*) to scavenge endogenous GlcN and confer tolerance to oxidative, drought, and cold stresses. Moreover, overexpression of *E. coli GlmS* in *Arabidopsis* promotes cell death at an early stage (Chu et al., 2010).

### 2.2 D-Glucosamine-6-phosphate N-acetyltransferase

The second enzyme in the HBP pathway is D-Glucosamine-6-phosphate N-acetyltransferase (GNA; EC 2.3.1.4), which converts GlcN-6-phosphate and acetyl-CoA to N-acetylglucosamine-6-phosphate (GlcNAc-6P) and CoA (**Figure 1**). GNA is a single-copy gene in the genome of most characterized organisms characterized. For example, the yeast *S. cerevisiae* gene (*YFL017C*) was demonstrated to exhibit GNA activity and is thus designated as *ScGNA1* (Mio et al., 1999). Additionally, the Arabidopsis genome also contains one *GNA* (*AtGNA*, At5g15770), the expression of which is ubiquitous in all organs (Riegler et al., 2012) and shows a slightly diurnal expression pattern (Usadel et al., 2008). In contrast to *Arabidopsis*, rice possesses two *GNA*s, including *OsGNA1* (LOC_Os09g31310) and *OsGNA* (LOC_Os02g48650). *OsGNA1* is highly expressed in root tissues (Jiang et al., 2005) but *OsGNA* is less characterized (Riegler et al., 2012) and has low expression levels in all tissues as revealed by the rice eFP browser (Jain et al., 2007). Based on the transient expression of the AtGNA-GFP fused protein in Arabidopsis protoplasts, its subcellular localization is primarily in the endoplasmic reticulum (ER) (Riegler et al., 2012), This result supports the role of UDP-GlcNAc, the end product of the HBP pathway, in protein glycosylation and synthesis of the GPI anchor in the ER. It was observed that deletion of yeast *ScGNA1* or *AfGNA1* and the loss-of-function of *AtGNA* by a T-DNA insertion (*AtGNA1-2* and *AtGNA1-3*), resulting in a complete block of GlcNAc production, is lethal (Mio et al., 1999; Riegler et al., 2012; Lockhart et al., 2020); this phenotype is similar to that obtained for the knockout mutants of *Arabidopsis* in GFAT, phospho-N-acetylglucosamine mutase or the double mutant *glcnac.ut1/glcnac.ut2* (Chen et al., 2014; Vu et al., 2019; Jia et al., 2023). This result also reflects the vital role of UDP-GlcNAc in plant growth. Although AtGNA has a low protein sequence identity to *Homo sapiens* HsGNA (~39.1%) and *S. cerevisiae* ScGNA (~35.0%), this protein crystal structure at 1.5 Å resolution exhibited very high structural similarity to these two orthologs (Riegler et al., 2012).

An EMS-mutagenized missense mutation in Arabidopsis *GNA*, known as *lignescens* (*lig*), causes plant growth defects and ectopic lignin accumulation under high temperature (28°C) conditions. Compared to the wild type, the *lig* mutant plants exhibit lower levels of UDP-GlcNAc than the wild type, accompanied by defects in N-linked protein glycosylation, ER stress, and unfolded protein response (UPR). Supporting evidence reveals the upregulation of *BiP3* expression, an ER stress marker, under high-temperature conditions and treatments with the ER stress-inducing agents, tunicamycin, and DTT, resulting in plants with phenotypes that mimic the *lig* mutant. Moreover, exogenous application of UDP-GlcNAc, GlcNAc, or GalNAc rescues the high-temperature sensitivity and ectopic accumulation of lignin observed in the *gna*/*lig* mutants. Thus, dysfunction of GNA causes a high-temperature-dependent defect in UDP-GlcNAc biosynthesis, which further affects N-linked protein glycosylation and lignin accumulation, mostly through the UPR (Nozaki et al., 2012).

The function of rice OsGNA1 was also reported by Jiang et al. (2005). *Osgna1* is a T-DNA insertion mutant that shows lower levels of UDP-GlcNAc and defects in N-linked protein glycosylation, as well as a reduction in O-linked glycosylation activity. The short-root phenotype of *Osgna1* is temperature-sensitive, particularly at 25°C, which can be largely rescued by a high temperature of 32°C. This low temperature-sensitive response in rice may be opposite to that of the Arabidopsis *Atgna* mutant, which shows greater sensitivity to high temperature. This discrepancy remains to be investigated in the future. These short roots observed in *Osgna1* are linked to defects in mitochondrial dehydrogenase activity, root viability, cell shape, and microtubule stability. The latter may result from a defect in O-linked glycosylation of microtubule-associated proteins (Jiang et al., 2005).

### 2.3 N-acetylglucosamine-phosphate mutase/phosphoacetylglucosamine mutase

N-acetylglucosamine-phosphate mutase (AGM; EC 5.4.2.3) or phosphoacetylglucosamine mutase catalyzes the isomerization of N-acetylglucosamine-6-P (GlcNAc-6-P) into N-acetylglucosamine-1-P (GlcNAc-1-P) (**Figure 1**). The growth of the yeast *ScAGM* deletion mutant (*Scagm*) cannot progress through five cell cycles. Overexpression of *ScAGM* may complement the growth defect of a phosphoglucomutase (PGM) double deletion mutant (*pgm1*/*pgm2*); however, overexpression of *ScPGM2*, a major *PGM*, cannot restore the growth of *Scagm1* deletion mutant cells. These data suggested that the different hexosephosphate mutases of *S. cerevisiae* share partially overlapping substrate specificities but they have distinct physiological functions (Hofmann et al., 1994). In mice, severely reduced AGM1/PGM3 activity causes lethality during embryonic development, whereas mutated mice with partial AGM1/PGM3 activity do not perish but display severe syndromes, such as sterility (Greig et al., 2007). Human patients with mutations in *PGM3*/*AGM1* will die in early infancy or have congenital immune system defects, developmental delays, and neurocognitive disorders (Ben-Khemis et al., 2017).

The Arabidopsis *AGM* gene (At5g18070) was first identified by selecting for complementation of *Escherichia coli* UV-sensitive mutants, and the identified gene was termed *DNA-DAMAGE-REPAIR/TOLERANCE 101* (*DRT101*). The N-terminus of AGM/DRT101 contains an amino acid region similar to the chloroplast transit peptide, suggesting its possible subcellular localization in chloroplasts (Pang et al., 1993). Arabidopsis AGM shares 38 to 44% amino acid identity with *Homo sapiens*, *S. cerevisiae*, and *Aspergillus fumigatus*, and their protein structures are highly conserved. Although two members of the Arabidopsis α-D-phosphoglucosamine mutase family, At5g17530 and At1g70820, are phylogenetically similar to AtAGM, only AtAGM functions in the isomerization of GlcNAc-1-P and GlcNAc-6-P. AtAGM has promiscuous substrates and catalyzes the interconversion of GlcNAc-1-P and GlcNAc-6-P and Glc-1-P and Glc-6-P; the catalytic reaction by AtAGM requires divalent cations, such as Mg^2+^ or Mn^2+^ (Jia et al., 2023).

Based on the RT-qPCR analyses, *AtAGM* is highly expressesed in the roots, flowers, and siliques, similar to the *AtGFAT* expression pattern. Moreover, unlike other HBP enzymes present in the cytosol or ER membrane surface, overexpression of the *35S::AGM-GFP* transgene in the *Atagm* background, i.e., *Atagm*-OE, reveals AtAGM localization in the cytosol, cytomembrane, chloroplasts, and mitochondria (Jia et al., 2023).

Similar to other HBP mutants, the homozygous knockout mutants, such as *Atagm2* (SAIL_187_F01) are lethal; however, the knockdown mutants, *Atagm1* (SALK_039132C) and *Atagm2* (+/-) can survive. The expression of the *AtAGM* gene in both *Atagm1* and *Atagm2* (+/-) is greatly reduced, and these mutants show a ~40% reduction in UDP-GlcNAc content compared to wild-type plants. Interestingly, overexpression of *AtAGM* in the *Atagm* background, i.e., *Atagm*-OE, does not increase UDP-GlcNAc contents; this likely results from feedback inhibition of UDP-GlcNAc, which affects the glutaminase function of GFAT (Olchowy et al., 2007; Walter et al., 2018; Vu et al., 2019). Thus, exogenous tunicamycin impairs UDP-GlcNAc inhibition and enhances AtGFAT activity, leading to increasingly higher levels of UDP-GlcNAc in *Atagm*-OE plants than in the wild-type plants (Jia et al., 2023). Thus, HBP is exquisitely regulated to maintain UDP-GlcNAc homeostasis, which plays a critical role in normal plant growth and development. Although these knockdown mutants display no conceivable phenotype, they show more vigorous growth than the wild type and *Atagm*-OE at maturity under normal growth conditions. This vigorous growth observed in the mutants presumably results from high chlorophyll contents that enhance photosynthetic capability. Moreover, these mutants show temperature-dependent (28°C) growth defects, including short roots and germination delay. Temperature-sensitive phenotypes can be abolished by exogenous UDP-GlcNAc (Jia et al., 2023). These data suggest that a small amount of UDP-GlcNAc is sufficient for normal plant growth, which is also observed in mouse embryonic fibroblasts (Boehmelt et al., 2000). However, plants need more UDP-GlcNAc when adapting to abiotic stress, and the mutant plants, such as *Atagm1* and *Atagm2* (+/-), cannot produce adequate UDP-GlcNAc under deleterious environments, leading to stress-induced growth defects.

**Figure 2:**
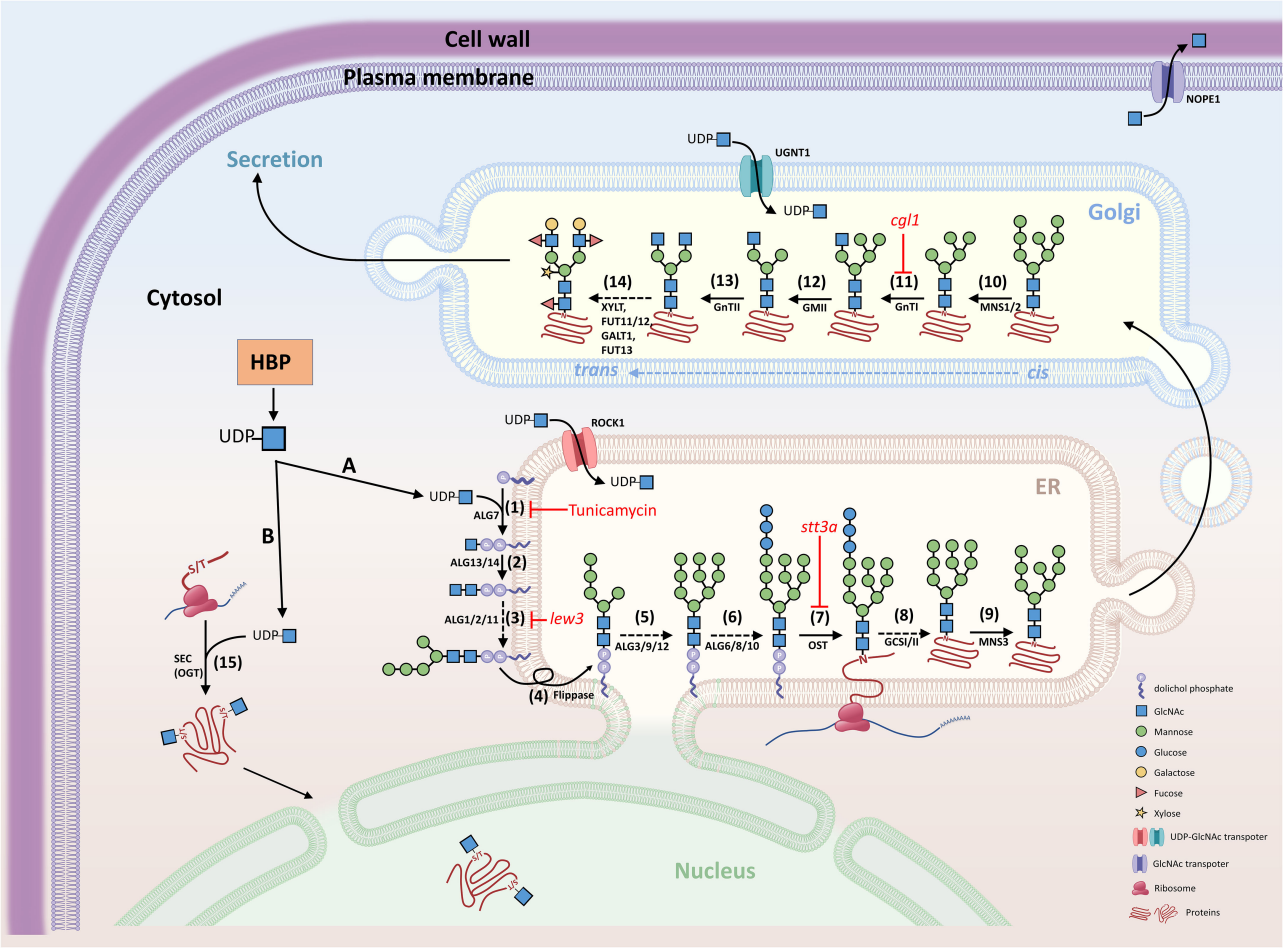
Schematic diagram of N-glycosylation and O-GlcNAcylation. GlcNAc is the fundamental amino sugar moiety essential for N-glycosylation and GlcNAcylation. **(A)** N-glycosylation. UDP-GlcNAc is generated by the hexosamine biosynthesis pathway (HBP) and provides GlcNAc for the initial biosynthesis of oligosaccharide precursors at the cytosolic side of the ER. The oligosaccharide precursor (Man5GlcNAc2-PP-Dol) enters the ER lumen for N-glycan modification and N-glycosylation of proteins. Complex and hybrid N-glycan processing occurs in the Golgi apparatus. Proteins with mature N-glycans will be secreted to their destinations. **(B)** O-GlcNAcylation. UDP-GlcNAc also provides the GlcNAc molecular unit directly to the Ser/Thr amino acids of proteins localized in the cytosol and nucleus. (1) Asparagine-linked glycosylation (ALG) enzyme ALG7, a UDP-N-acetylglucosamine:dolichol phosphate N-acetylglucosamine-1-P transferase; (2) ALG13 and ALG14, UDP-N-acetylglucosamine transferase subunits; (3) ALG1/2/11, mannosyltransferases; (4) Flippase-like protein; (5) ALG3/9/12, mannosyltransferases; (6) ALG6/8/10, glucosyltransferases; (7) OST, oligosaccharyltransferase complex; (8) GCSI/II, glucosidases; (9) MNS3, ER-α-mannosidase I; (10) MNS1/2, Golgi-α-mannosidase I; (11) GnTI, β-(1->2)-N-acetylglucosaminyltransferase I or COMPLEX GLYCAN LESS 1 (CGL1); (12) GMII, Golgi α-mannosidase II; (13) GnTII, β-(1->2)-N-acetylglucosaminyltransferase II; (14) XYLT, β-(1->2)-xylosyltransferase; FUT11/12, core α-(1->3)-fucosyltransferases; GALT1, β-(1->3)-galactosyltransferase 1; FUT13, α-(1->4)-fucosyltransferase; (15) SEC, SECRET AGENT (O-GlcNAc transferase, OGT); ROCK1, REPRESSOR OF CYTOKININ DEFICIENCY 1; UGNT1, UDP-GlcNAc transporter; NOPE1, NO PERCEPTION 1. The nomenclature of enzymes is generally based on the report by Strasser et al. (2021).

Total protein blots stained with concanavalin A (ConA) lectin revealed that glycoproteins show no significant difference, whereas the N-glycan composition varies among wild type, *Atagm2* (+/-), and *Atagm*-OE. Moreover, an obvious impairment of O-GlcNAcylation is observed in the *Atagm* mutants. The temperature-sensitive growth defects are primarily linked to the impairment of protein O-GlcNAcylation but not N-glycosylation because the O-GlcNAcylation deficient mutants *Atsec*s, in which O-GlcNAc transferase (OGT, **Figure 2**) is defective, also display temperature-sensitive phenotypes; however, no significant phenotype was observed in the N-glycosylation deficient mutant *Atstt3a*, in which oligosaccharyltransferase (OST, **Figure 2**) is defective (Jia et al., 2023).

### 2.4 N-acetylglucosamine-1-P uridylyltransferase/or UDP-N-acetlyglucosamine-1-P pyrophosphorylase

The last reaction of HBP is the uridylation of GlcNAc-1-P into UDP-GlcNAc by N-acetylglucosamine-1-P uridylyltransferase (GlcNAc1pUT), named after a forward catalytic reaction (Yang et al., 2010) or UDP-N-acetylglucosamine-1-P pyrophosphorylase (UAP; EC 2.7.7.23), named after a reverse reaction (**Figure 1**). Although the biosynthesis of UDP-GlcNAc in prokaryotes and eukaryotes is similar, their catalytic specificity is diverse (Mengin-Lecreulx and Van Heijenoort, 1993). In bacteria, GlmU is a bifunctional enzyme that exhibits both the phosphoglucosamine acetyltransferase and UDP-N-acetylglucosamine pyrophosphorylase activities; however, these two enzymatic activities are encoded by distinct essential genes in eukaryotes. Thus, GlmU catalyzes the acetylation of GlcN-1-P into GlcNAc-1-P followed by the uridylation of GlcNAc-1-P into UDP-GlcNAc (**Figure 1**, green dashed line). Inactivation of the *GlmU* gene reduces glycoprotein synthesis, leading to changes in cell shape and lysis changes (Mengin-Lecreulx and Van Heijenoort, 1993). In yeast, a null mutation of yeast UAP1/QRI1 is lethal, which mainly shows swollen and lysed cell shapes (Mio et al., 1998). In *Drosophila melanogaster*, the *cabrio*/*mummy* mutant derived from EMS mutagenesis loses *DmUAP* function and exhibits defects in dorsal closure, central nervous system, and embryo development (Schimmelpfeng et al., 2006). Two human UAPs (AGX1 and AGX2) were identified with only a 17-amino acid difference and these UAPs were derived from alternative splicing and led to preferential substrate specificity in GalNAc-1-P and GlcNAc-1-P, respectively (Wang-Gillam et al., 1998; Peneff et al., 2001).

In *Arabidopsis*, two UAPs termed GlcNAc1pUT1 and GlcNAc1pUT2 are encoded by *GlcNA.UT1* and *GlcNA.UT2*, respectively. They were first cloned and their biochemical specificity was characterized by Yang et al. (2010). In general, GlcNAc1pUT1 uses GlcNAc-1-P or GalNAc-1-P as substrates together with UTP to form UDP-GlcNAc or UDP-GalNAc and PPi. This uridylation activity is similar to that of human AGX1. GlcNAc1pUT2 has broader substrate specificities and may utilize Glc-1-P as a substrate in addition to GlcNAc-1-P and GalNAc-1-P. Thus, the enzymatic activity of AtGlcNAc1pUT2 is closer to that of yeast UAP1/QRI1 (Mio et al., 1998) and rice OsUAP1/SPL29 (Wang et al., 2015). The substrate specificity between GlcNAc1pUT1 and GlcNAc1pUT2 is likely related to their protein structures, which share a similar fold but vary in some loop regions. The biochemical assay also indicated that Arabidopsis GlcNAc1pUTs require divalent ions (such as Mg^2+^ or Mn^2+^) for their enzymatic activity. Gel-filtration analysis revealed the monomer structure of the native GlcNAc1pUT1 protein (Yang et al., 2010), which is different from the dimer structure of human AGX1 (Wang-Gillam et al., 1998) and yeast UAP1/QRI1 (Milewski et al., 2006). Although GlcNAc1pUT1 and human AGX1 share 32% protein sequence identity, their three-dimensional protein structure models display a conserved catalytic fold and key conserved motifs (Yang et al., 2010).

AtGlcNAc1pUT1 may utilize both UDP-GlcNAc and UDP-GalNAc as substrates (Yang et al., 2010). UDP-GalNAc has been found in several plant species, including squash (Tolstikov and Fiehn, 2002) and dahlia tubers (Gonzalez and Pontis, 1963). In barley, UDP-Glc 4-epimerase or UDP-Gal 4-epimerase (UGE; EC 5.1.3.2) catalyzes the interconversion of UDP-Glc and UDP-Gal; the enzyme can also reversibly catalyze UDP-GlcNAc and UDP-GalNAc (Zhang et al., 2006). Although several UGE genes have been cloned in plants, such as peas, *Arabidopsis*, and the endospermous legume guar (Dörmann and Benning, 1996; Lake and Slocum, 1998; Joersbo et al., 1999), the GalNAc targets of glycans and glycoproteins and their physiological significance remain to be further examined. As UDP-GlcNAc and UDP-GalNAc contain the same molecular mass, they cannot be distinguished by mass spectrometry analysis. Specific HPLC analysis can separate these two hexosamines obtained from Arabidopsis tissues (Nozaki et al., 2012). In general, UDP-GlcNAc is more abundant than UDP-GalNAc in plant tissues under normal or temperature-stress conditions (Nozaki et al., 2012).

Mutation of Arabidopsis *GlcNAc.UT1* or *GlcNAc.UT2* shows no conceivable phenotype, whereas the double mutant is lethal (Chen et al., 2014), reflecting functional redundancy and the pivotal role of these genes in normal plant growth and development. The heterozygous double mutant *GlcNA.UT1*/*glcna.ut1 glcna.ut2*/*glcna.ut2* obtained from the F2 segregating population following reciprocal crosses of *glcna.ut1* and *glcna.ut2*, displays sterility. Furthermore, this heterozygous double mutant reveals the aberrant transmission of (*glcna.ut1*, *glcna.ut2*) gametes, which is consistent with the defects in male gametophytes during late vacuolation (or pollen mitosis I stage) and in female gametophytes during the uninucleate embryo sac stage. Interestingly, one normal allele of *GlcNA.UT2* in the *glcna.ut1*/*glcna.ut1 GlcNA.UT2*/*glcna.ut2* mutant has normal gamete transmission of (*glcna.ut1*, *glcna.ut2*) and gametophytic development, except that the development of numerous embryos is arrested during the early globular stage (Chen et al., 2014). Thus, GlcNA1pUT1 and GlcNA1pUT2 differentially regulate gametophytic and embryonic development, which may be associated with their spatiotemporal expression, subtle difference in GlcNAc1pUTase activity, and metabolic complementation (Bonhomme et al., 1998). To further study Arabidopsis *GlcNA.UT* function, the RNAi transgenic plants, termed iU1s, were generated by RNA interference of *GlcNA.UT1* expression in the *glcna.ut2* null mutant background. The iU1 transgenic plants resemble the heterogeneous double mutant *GlcNA.UT1*/*glcna.ut1 glcna.ut2*/*glcna.ut2* showing sterility under normal growth conditions. The iU1s possess normal levels of hexosamine (UDP-GlcNAc and UDP-GalNAc) compared to the wild type under normal growth conditions, whereas they show reduced hexosamine biosynthesis, altered protein N-glycosylation, and an unfolded protein response under salt-stressed conditions. Moreover, the iU1s confer slat hypersensitivity, including delay of seed germination and early seedling establishment, in association with the induction of ABA biosynthesis and its signal networks under salt stress. Furthermore, microarray analysis data support the upregulation of genes involved in ABA (such as *NCED3*, *ABI5*, and *ABCG25*) and salt stress responses (such as *RD29A*, *RD29B*, and *DREB2A*) (Chen et al., 2022).

#### 2.4.1 Biochemical variations and UDP-GlcNAc transport

Likewise, rice *GlcNA.UT*s termed UAP1 or SPOTTED LEAF 29 (SPL29) and UAP2 (Wang et al., 2015, Wang et al., 2021) can catalyze GlcNAc-1-P and GalNAc-1-P as substrates to form UDP-GlcNAc (Xiao et al., 2018; Wang et al., 2021). OsUAP1/SPL29 irreversibly catalyzes the decomposition of uridine 5’-diphosphoglucose (UDPG) to form UTP and Glc-1-P. The loss-of-function *Osuap1*/*spl29* mutant accumulates UDPG, which may be involved in ROS accumulation, early leaf senescence, plant cell death (PCD), and leaf lesion mimics (or defense response) (Wang et al., 2015; Xiao et al., 2018). It remains unknown whether Arabidopsis GlcNAc1pUTs may use UDPG as a substrate similar to rice. As spotted leaves are a lesion-mimic phenotype of the hypersensitive response, the *Osuap1*/*spl29* mutant causes induction of the defense response by upregulation of defense-responsive genes and bacterial blight resistance. In addition, early leaf senescence and defense response enhancement are linked to the accumulation of jasmonic acid, abscisic acid, and reactive oxygen species (ROS) in O*suap1*/*spl29* mutant plants (Wang et al., 2015). *OsUAP2* overexpression may rescue *Osuap1*/*spl29* mutant phenotypes, reflecting that they share functional redundancy. *OsUAP2* is primarily expressed in the early leaf development and *OsUAP1/SPL29* at the whole leaf developmental stages, and both genes synergistically regulate rice leaf development and protect them from early senescence (Wang et al., 2021). Thus, unlike Arabidopsis single mutant *glcna.ut1* or *glcna.ut2*, which show no conceivable phenotype, the single *Osuap1*/*spl29* mutant displays early senescence and lesion-mimic spotted leaves, presumably indicating that rice plants are more sensitive to the defense response. The functions of HBP-related genes/proteins are summarized in **Table 1**.

**Table 1:** Summary of HBP-related gene functions.

Enzyme	Other names	Substrates	Products	Localization	Stress	Mutant phenotypes
Glutamine:Fru-6-P amidotransferase (GFAT) (A13g24090)	GFAT, GlcN6P synthase (eukaryotes); Glms (prokaryotes); GFA (lower eukaryotes)	D-Fru-6-P L-glutamine	D-GlcN-6-P L-glutamate	Cytoplasm^2^	Oxidation, drought, cold	Inactive pollen activity, reduced GlcN, UDP-GlcNAc, and protein N-glycosylation; impaired plant development and partial sterility; increased tunicamycin sensitivity
GlcN-6P acetyltransferase (GNA) (At5g15770)	GNA1 (yeast, rice) GNA LIG (Arabidopsis)	D-GlcN-6-P acetyl-CoA	GlcNAc CoA	ER	Arabidopsis: sensitive to high temperature (28 °C) rice: sensitive to low temperature (25°C)	Arabidopsis: reduced UDP-GIcNAc, protein N-glycosylation, and O-GlcNAcylation activity; induced ER stress and UPR; rice: temperature-dependent root elongation and lignin deposition
N-acetylglucosamine-phosphate mutase (AGM) or phosphoacetylglucosamine mutase (A15g18070)	AGM, DRT101 (Arabidopsis); AGM1/PGM3 (mice)	GlcNAc-6-P Glc-6-P	GlcNAc-1-P Glc-1-P	Cytosol, cytomembrane, chloroplast, mitochondrium	Temperature	Arabidopsis: vigorous growth, reduced UDP-GIcNAc; high temperature-dependent (28°C) growth defects, including short roots and germination delay; impairment of O-GlcNAcylation
N-acetylglucosamine-1-P uridylyltransferase (GlcNAc1pUT); UDP-N- acetylglucosamine pyrophosphorylase (UAP) (AT1G31070, AT2G35020)	GlcNAc1pUT1 and 2 (Arabidopsis); UAP1, SPL29 (rice); GlmU (prokaryote); UAP1, QRI1 (yeast); AGX1, AGX2 (human)	GlcNAc-1-P UDPG^3^	UDP- GlcNAc UDP- GalNAc Glc-1-P	Cytoplasm, plasma membrane	Arabidopsis: salt-sensitive, response to UV rice: bacterial blight resistance, defense response, sensitivity to high temperature	Arabidopsis: defective in gametogenesis and embryo development; salt-induced delay of seed germination and early seedling growth; reduced UDP-hexoNAc, altered N-glycosylation, and induced UPR under salt stress rice: leaf senescence and defense response, UDPG and ROS accumulation, short root and germination delay at high temperature, reduced N-glycosylation

^1^Mutant phenotypes represent knockdown mutant plants because the knockout mutants are lethal.

^2^Localization of GFAT and GlcNAc1pUTs is based on the annotation of The Arabidopsis Information Resource (TAIR).

^3^UDPG, uridine 5’-diphosphoglucose.

It was reported that NO PERCEPTION 1 (NOPE1) acts as the GlcNAc transporter localized in the plasma membrane of the root tissues of rice and maize. NOPE1 transports GlcNAc into the rhizosphere, where it serves as a molecular signal to enhance branching hyphae of arbuscular mycorrhiza (AM) and benefit the symbiosis between AMs and host plants (Nadal et al., 2017). Arabidopsis genome contains UDP-GlcNAc transporters, one was termed UDP-GlcNAc transporter (UGNT1; At4g32272), in the Golgi membrane, which transports UDP-GlcNAc from the cytosol to the Golgi to initiate complex glycan processing. The *Atugnt1* null mutant plants lack complex and hybrid N-glycans, and the N-glycopeptides primarily contain high-mannose structures. Moreover, AtUGNT1 is also needed for the biosynthesis of GlcNAc-containing glycosyl inositol phosphorylceramides (GIPCs) (Ebert et al., 2018). Another transporter for UDP-GlcNAc and UDP-GalNAc is the REPRESSOR OF CYTOKININ DEFICIENCY 1 (ROCK1, At5g65000), which is localized in the ER membrane and involved in ER quality control. The *Atrock1* mutant reduces the activity of cytokinin oxidases/dehydrogenases (CKXs, cytokinin-degrading enzymes) and impairs the cytokinin-deficient mutant phenotype. Although the N-glycosylation of CKX1 is not affected in *Atrock1*, the stability of CKX1 is enhanced in the mutant (Niemann et al., 2015) (**Figure 2**). The function of ROCK1 in providing UDP-GlcNAc for the ER lumen remains unknown because GlcNAc conjugates in the ER have not been uncovered to date. One possibility is that UDP-GlcNAc in the ER may be transported to the Golgi apparatus for complex glycan modification (Ebert et al., 2018).

## 3 Salvage pathway of GlcN and GlcNAc

In addition to *de novo* biosynthesis through HBP, UDP-GlcNAc can be generated by the salvage pathway. In mammals, GlcN and GlcNAc can be retrieved from environmental resources and the degradation of glycans or glycoconjugates. These salvage nutrients can be used as dietary supplements to benefit from the treatment of disorders linked to glycosylation. GlcN can be phosphorylated to form GlcN-6-P by hexokinase and then enters the HBP to produce UDP-GlcNAc (**Figure 1**, purple line) (Kornfeld, 1967; Krug et al., 1984). In Arabidopsis, the exogenous application of GlcN to *Atgfat1* mutant plants may rescue mutant phenotypes, indicating that the GlcN can be converted into GlcN-6-P, which further enters HBP to form UDP-GlcNAc (Vu et al., 2019).

GlcNAc can be phosphorylated to form GlcNAc-6-P by GlcNAc kinase (GNK) or N-acetylglucosamine kinase (NAGK) (Gindzieński et al., 1974; Allen and Walker, 1980; Hinderlich et al., 2000; Berger et al., 2002; Ryczko et al., 2016). This intermediate GlcNAc-6-P further enters the HBP to form UDP-GlcNAc (**Figure 1**, purple line). In mammals, NAGK is needed for embryonic mouse development (Dickinson et al., 2016). Deletion of NAGK increases *de novo* hexosamine biosynthesis; conversely, glutamine deprivation inhibits *de novo* HBP but triggers the NAGK-dependent salvage pathway in pancreatic ductal adenocarcinoma (PDAC) (Campbell et al., 2021), suggesting that cross-talk occurs between the salvage and *de novo* HBP. In higher plants, the GNK was first identified and characterized in *Arabidopsis* by Furo et al. (2015). Arabidopsis GNK (At1g30540) and human NAGK proteins share high structural conservation, particularly in GlcNAc and ATP binding domains. The kinase activity of AtGNK was confirmed by an enzymatic activity assay *in vitro* through recombinant AtGNK protein. Substrate analysis further supports that AtGNK exhibits high specificity for GlcNAc and less specificity for GalNAc. Furthermore, although the null mutant *Atgnk* shows no conceivable phenotype, the mutant plants reveal lower levels of UDP-GlcNAc than the wild type and are insensitive to the exogenous application of GlcNAc (Furo et al., 2015). The GlcNAc salvage pathway is also observed in the *Atgna*/*lig* mutant, which is defective in the conversion of GlcN-6-P to GlcNAc-6-P and leads to a reduction in UDP-GlcNAc levels, high-temperature sensitivity, and ectopic accumulation of lignin. Exogenous application of GlcNAc rescues the *Atgna*/*lig* mutant phenotypes and increases the UDP-GlcNAc content (Nozaki et al., 2012). Therefore, the *Atgna*/*lig* mutant fails to convert GlcN6-P to GlcNAc-6P; however, exogenous GlcNAc can be catalyzed by AtGNK to form GlcNAc-6-P, which further enters the HBP to produce UDP-GlcNAc. Similarly, wild-type plants (Col-o) produce more UDP-HexNAc (UDP-GlcNAc and UDP-GalNAc) by exogenous GlcNAc under normal and salt-stressed conditions (Chen et al., 2022). The coexistence of HBP and salvage pathways may finetune the homeostasis of UDP-GlcNAc contents in plants in response to nutrient fluctuations and environmental stimuli.

## 4 N-linked glycosylation

Asparagine (Asn- or N-) glycosylation is among the most common co- or post-translational modifications, which is essential for plant growth and stress responses and is conserved across eukaryotes (Banerjee et al., 2007; Bao and Howell, 2017; Nagashima et al., 2018). N-glycosylation regulates protein folding, transport, sorting, degradation, and intracellular signaling (Helenius and Aebi, 2001; Molinari, 2007; Aebi, 2013; Lannoo and Van Damme, 2015; Shin et al., 2018). Most secreted and membrane-associated proteins are N-glycosylated proteins (N-glycoproteins), and they are involved in a wide range of cellular processes, including cell wall biosynthesis (Jose-Estanyol and Puigdomenech, 2000), pollination (Hancock et al., 2005), pathogen defense (Pearce et al., 2007), and cell-to-cell communication (Taoka et al., 2007). Biosynthesis of N-glycan occurs in multiple subcellular compartments, including the cytosol, endoplasmic reticulum (ER) lumen, and Golgi apparatus (Pattison and Amtmann, 2009). Initially, N-glycan is formed as an oligosaccharide precursor on a lipid-linked carrier, dolichol pyrophosphate (PP-Dol), on the cytosolic side of the ER membrane (**Figure 2**). Two GlcNAc molecules are first transferred to PP-Dol by GlcNAc-1-phosphotransferase (GPT) or asparagine-linked glycosylation (ALG) enzyme ALG7 and the ALG13/14. Subsequently, five mannose (Man) residues are added by mannosyltransferases, ALG1/2/11, to form Man5GlcNAc2-PP-Dol (Burda and Aebi, 1999; Strasser et al., 2021). This oligosaccharide precursor is then flipped to face the ER lumen for further modification (Pattison and Amtmann, 2009; Strasser et al., 2021).

In the ER lumen, four more Man and three Glc residues are sequentially added to form the core oligosaccharide Glc3Man9GlcNAc2-PP-Dol, which is assembled by a series of membrane-bound mannosyltransferases (ALG3/9/12) and glycosyltransferases (ALG6/8/10) (Snider et al., 1980; Helenius and Aebi, 2001; Nagashima et al., 2018; Strasser et al., 2021). N-glycosylation occurs in the ER lumen by transferring the core oligosaccharide to Asn in the Asn-X-Ser/Thr motif (X, any amino acid except Pro) of a nascent peptide, which is mediated by an oligosaccharyltransferase (OST) complex (Burda and Aebi, 1999; Pattison and Amtmann, 2009; Strasser, 2016). The N-linked Glc3Man9GlcNAc2 glycan is further processed by the sequential removal of three Glc residues by glucosidase I and II (GCSI and GCSII) (Trombetta and Parodi, 2003; Nagashima et al., 2018; Strasser et al., 2021), and a Man residue is removed by the ER-α-mannosidase I (MNS3; Liebminger et al., 2009). The correctly folded glycoproteins leave the ER and move into the Golgi apparatus for further complex and hybrid N-glycan modification (Strasser, 2016).

In the Golgi, the first N-glycan processing is carried out by α-1,2-mannosidase I (MNS1/2), which removes three Man residues from Man8GlcNAc2 to form Man5GlcNAc2, the product for the subsequent complex and hybrid N-glycan processing. The formation of complex and hybrid N-glycan is initiated by the N-ACETYLGLUCOSAMINYL TRANSFERASE I (GnTI)-mediated addition of the GlcNAc residue to the α-1,3-linked Man of the Man5GlcNAc2 to form GlcNAcMan5GlcNAc2 (von Schaewen et al., 1993; Strasser et al., 1999). Subsequently, alternative processing pathways can occur in plants (Bencúr et al., 2005). In the canonical pathway, two Man residues are cleaved from GlcNAcMan5GlcNAc by Golgi-α-mannosidase II (GMII), followed by GnTII-mediated addition of another GlcNAc residue to the α1,6-linked Man to form GlcNAc2Man3GlcNAc2. Afterward, Xyl, Fuc, and two Gal are added to the acceptor substrate GlcNAc2Man3GlcNAc2, which are catalyzed by XylT (xylosyltransferase), FUT11/12 (fucosyltransferases), and GALT1 (galactosyltransferase), respectively. Finally, FUT13 (α-(1->,4)-fucosyltransferase) transfers a Fuc residue to the α-(1->4)-linked GlcNAc to complete the Lewis A-type structure, which is a trisaccharide structure (**Figure 2**) (Strasser, 2016; Strasser et al., 2021). The resulting products could be secreted to their destinations. Golgi-resident GnTI is the key enzyme in complex and hybrid N-glycan processing. The Arabidopsis *complex glycan less 1* (*cgl1*) mutant, which is defective in GnTI activity, lacks complex and hybrid N-glycans and exhibits reduced N-glycosylation efficiency (von Schaewen et al., 1993; Strasser et al., 2005; Frank et al., 2008; Farid et al., 2013). However, the *cgl1* mutant displays no apparent phenotype under normal growth conditions but confers salt hypersensitivity (Kang et al., 2008). In contrast to the Arabidopsis *cgl1* mutant, the rice *gnt1* mutant displays severe phenotypes showing arrest in postseedling development, defects in cell wall biosynthesis, and reduced cytokinin signaling (Fanata et al., 2013). The mechanisms that cause these markedly different phenotypes between Arabidopsis *cgl1* and rice *gnt1* remain to be illustrated in the future.

Interruption with N-glycan biosynthesis at any step by mutation of genes or treatments of pharmaceutical drugs, such as tunicamycin and DTT, will lead to incomplete N-glycans and affect N-glycosylated proteins (Pattison and Amtmann, 2009). Unfolded or misfolded proteins will accumulate in the ER and result in ER stress; eventually, the unfolded protein response (UPR) is activated to enhance the capacity for protein folding, increase the ER quality control, impair general protein translation, and maintain ER homeostasis (Bao and Howell, 2017; Yu et al., 2022). Defects in N-glycan processing may impair plant growth and stress responses or cause lethality (Lane et al., 2001; Koiwa et al., 2003; Lerouxel et al., 2005; Zhang et al., 2009; Fanata et al., 2013; Bao and Howell, 2017; Nagashima et al., 2018). Despite the significance of N-glycosylation, most studies in the past have focused on the core N-glycan formation in the ER lumen and the modification of complex N-glycans on glycoproteins in the Golgi apparatus. The effect of cytosolic oligosaccharide precursor production on plant growth and stress response is less addressed. As mentioned above, HBP generates UDP-GlcNAc to provide GlcNAc donors and initiate oligosaccharide precursor production on the cytosolic side of the ER. Defects in HBP enzymes may reduce UDP-GlcNAc levels, impair N-linked glycosylation, and alter plant growth under normal (Wang et al., 2015; Xiao et al., 2018) or abiotic stress conditions (Zhang et al., 2009; Nozaki et al., 2012; Chen et al., 2022). Moreover, a complete block of HBP normally leads to lethality (Chen et al., 2014; Vu et al., 2019; Jia et al., 2023).

It has been reported that N-glycan processing mutants alter the abiotic stress responses, such as salt stress. The *staurosporine and temperature sensitive 3a* (*stt3a*) mutant, in which a catalytic subunit of the OST complex in the ER is defective, and *leaf wilting 3* (*lew3*), a mutant that lacks α-1,2-mannosyltransferase, induce UPR-mediated *BiP* gene expression and enhance salt stress sensitivity (Koiwa et al., 2003; Zhang et al., 2009; Jiao et al., 2020). However, Arabidopsis *complex glycan 1* (*cgl1*), a mutant that lacks GnTI activity, shows a deprived complex and hybrid N-glycans and confers salt hypersensitivity (Frank et al., 2008; Kang et al., 2008). Unlike *stt3a*, which shows a UPR response, the *cgl1* mutant fails to induce a UPR response. Thus, the UPR is likely not the major player that enhances salt hypersensitivity in the mutants with defective N-glycan processing. Furthermore, the mutation of *UDP-GlcNAc transporter 1* (*UGNT1*) leads to deprived complex and hybrid N-glycan in the Golgi apparatus and does not increase salt hypersensitivity (Ebert et al., 2018). These data suggest that mature complex N-glycans are not the major factor leading to salt hypersensitivity. It was generally proposed that mutants defective in N-glycan processing in the ER lumen or Golgi apparatus might alter a different set of glycoprotein and/or glycolipid functions, which further integrate to alter plant growth and abiotic stress response. Compared to the Arabidopsis *stt3a* mutant showing short root elongation under salt stress, *glcna.ut* mutants, such as the RNAi knockdown mutants iU1s, display normal root elongation under salt stress (Chen et al., 2022). Although the UPR response is induced and N-linked glycosylation is impaired in iU1 mutants, these mutants exhibit salt hypersensitivity in terms of delayed seed germination and early seedling establishment, the phenotypes of which are different from those of *stt3a* mutant plants. The *stt3a* mutant, such as *stt3a-2*, also displays a higher stomatal density and transpiration rate in association with low endogenous ABA and auxin (IAA) levels. Thus, *stt3a* mutant plants are more sensitive to salt and drought stresses. These mutant phenotypes are correlated with the underglycosylation of β-glucosidase (AtBG1), catalyzing the conversion of conjugated ABA or IAA to its active form (Jiao et al., 2020). Consistently, exogenous application of ABA or IAA to *stt3a-2* may partially rescue the mutant phenotypes. In contrast, the *GlcNA.UT* knockdown lines iU1s, reveal higher levels of ABA under salt stress conditions (Chen et al., 2022). Thus, although *stt3a* and iU1 affect the N-glycosylation of proteins, they could use different mechanisms in response to salt stress. It is conceivable that GlcNAc1pUTs produce UDP-GlcNAc not only for N-glycan synthesis in the ER lumen and maturation in the Golgi apparatus but also for the O-GlcNAcylation of primarily cytosolic and nuclear proteins (**Figure 2**). Thus, in addition to N-glycan processing, HBP has a wider range of effects on plant growth and abiotic stress response through diverse GlcNAc targets or conjugates.

## 5 O-GlcNAcylation

O-GlcNAcylation is the addition of O-linked N-acetylglucosamine (O-GlcNAc) to the serine (Ser) and threonine (Thr) residues of nucleocytoplasmic and mitochondrial proteins (Hu et al., 2009; Ma et al., 2022), which was first reported by Torres and Hart (1984). In contrast to N-linked glycosylation, which involves the attachment of complex glycans to proteins for the secretary pathway, O-GlcNAcylation involves the direct addition of a single GlcNAc residue to the Ser/Thr residues of proteins, which primarily occurs in the cytosol or nucleus (**Figure 2**). O-GlcNAcylation is also among the most common co- or post-translational modifications and is conserved across organisms (Joshi et al., 2018; Ma et al., 2022). O-GlcNAcylated proteins are involved in most aspects of cellular functions including metabolism, transcriptional regulation, signaling, cell cycle regulation, protein trafficking, protein-protein interaction, and cell structure (Wells et al., 2001; Love and Hanover, 2005; Hart et al., 2011; Liu et al., 2022). In mammals, dysregulation of O-GlcNAcylation may be linked to chronic disorders, including the occurrence and progression of cancer (Slawson and Hart, 2011; Singh et al., 2015), diabetic complications (Peterson and Hart, 2016), neurodegeneration (Hart et al., 2011; Gong et al., 2012), and cardiovascular diseases (Wang et al., 2023), and the immune system (Golks and Guerini, 2008). Thus, manipulating O-GlcNAcylation may be a potential strategy for cancer therapy (Lu et al., 2022).

O-GlcNAcylated proteins are usually phosphorylated. As O-GlcNAcylation and phosphorylation are dynamic reactions that cycle rapidly, both post-translational modifications compete with the same Ser/Thr sites or modify nearby/or distant sites to show complex interplay and coordinate protein stability and function in response to external stimuli (Slawson and Hart, 2003; Wang et al., 2008c; Butkinaree et al., 2010; Zeidan and Hart, 2010; Hart et al., 2011; Martínez-Turiño et al., 2018; Xu et al., 2019). For instance, O-GlcNAcylation and phosphorylation coexist in the capsid protein (CP) of the plum pox virus (PPV). Although O-GlcNAcylation of PPV CP is not needed for virus viability, it increases viral infection (Pérez Jde et al., 2013; Martínez-Turiño et al., 2018). Moreover, vernalization increases the O-GlcNAc modification of nuclear TaGRP2 (a repressor in vernalization) and the phosphorylation of VER2 (an activator in vernalization); both modified proteins antagonistically regulate the expression of TaVRN1 to mediate flowering in winter wheat (Xiao et al., 2014; Xu et al., 2019).

The first public bioinformatics resource of O-GlcNAcylated proteins was established by Wang et al. (2011), in which approximately 1240 proteins are potentially O-GlcNAcylated. Later, over 1000 O-GlcNAcylated proteins were uncovered in different studies of mammalian cells (Trinidad et al., 2012; Hahne et al., 2013). Recently, with more improved techniques, over 5000 O-GlcNAcylated proteins were identified using human models (Wulff-Fuentes et al., 2021). In *Arabidopsis*, Xu et al. (2017) identified 262 proteins with O-GlcNAcylation. Among them, the O-GlcNAcylated and O-fucosylated protein AtACINUS is involved in ABA sensitivity through alternative splicing of HIGH LEVEL OF BETA-AMYLASE ACTIVITY 1 (HBA1) and ABA HYPERSENSITIVE 1 (ABH1), negative regulators of ABA signaling, and in flowering through transcriptional regulation of the floral repressor FLOWERING LOCUS C (FLC) (Bi et al., 2021). In addition, a total of 168 O-GlcNAcylated proteins were found in winter wheat (Xu et al., 2019); these proteins perform functions in metabolism, response to stimuli, cellular processing, signal transduction, and transcriptional regulation. Thus, the total number of identified proteins of O-GlcNAcylation is far lower in plants than in mammalian cells.

O-GlcNAcylation is catalyzed by O-GlcNAc transferase (OGT) (**Figure 2**). Phylogenetic analysis revealed that metazoans contain a single OGT, whereas vascular plants and moss have two homologs of OGTs (Olszewski et al., 2010). Considering that GlcNAc is needed for O-GlcNAcylation and UDP-GlcNAc, the donor of GlcNAc, is synthesized through HBP, HBP might perform crosstalk with O-GlcNAcylation to optimize nutrient status and O-GlcNAcylation cycling. In *Drosophila*, protein O-GlcNAcylation displays a circadian rhythm mediated by the HBP enzyme GFAT and the O-GlcNAcylation enzymes, OGT and O-GlcNAcase (OGA), an enzyme removing GlcNAc from O-GlcNAcylated proteins (Liu et al., 2021). The Arabidopsis knockdown mutant *Atagm* reduces UDP-GlcNAc production and shows a temperature-dependent growth defect that is associated with the impairment of protein O-GlcNAcylation (Jia et al., 2023). In *Arabidopsis*, two OGT homologs, SECRET AGENT (SEC) and SPINDLY (SPY), catalyze O-GlcNAcylation and O-linked fucosylation, respectively (Hartweck et al., 2002; Zentella et al., 2016, Zentella et al., 2017). The Arabidopsis null *sec* mutant only displays a subtle phenotype (Hartweck et al., 2002), but the *spy* mutant shows an apparent GA response, indicating that SPY acts as a negative regulator of GA signaling (Wilson and Somerville, 1995; Jacobsen et al., 1996). Moreover, the *sec*/*spy* double mutant is lethal, with defects in gamete and seed development that are similar to the knockout *OGT* mutants in mice and *Drosophila*, in which embryonic lethality occurs (Shafi et al., 2000; Gambetta et al., 2009). These data indicate that although SEC and SPY have overlapping functions involved in GA signaling, they also have distinct roles and may play a synergistic function in plant growth and development (Hartweck et al., 2002, Hartweck et al., 2006; Zentella et al., 2016, Zentella et al., 2017). Later, it was reported that the Arabidopsis *sec* mutant displays an early-flowering phenotype, which is associated with the inhibition of O-GlcNAcylation of ARABIDOPSIS HOMOLOG OF TRITHORAX1 (ATX1), a histone lysine methyltransferase (HKMT). The impaired activity of ATX1 reduces histone H3 lysine 4 trimethylation (H3K4me3) of the *FLC* gene, a negative regulator of flowering (Xing et al., 2018). The Arabidopsis DELLA protein RGA (REPRESSOR OF *ga1-3*), a master negative regulator of the GA response, is O-GlcNAcylated by SEC; this suppresses the interactions of RGA with other key transcription factors, such as PIFs, BZR1, and JAZ1, which are involved in light, brassinosteroid, and jasmonate signalings, respectively (Zentella et al., 2016). In addition to DELLA proteins, several important transcription factors involved in plant hormone signaling are O-GlcNAcylated, such as ARFs, TCPs, EIN2, and ABF3, which are involved in the signaling of auxin, cytokinin, ethylene, and ABA, respectively (Xu et al., 2017). Compared to mammals, numerous proteins of O-GlcNAcylation in plants remain to be uncovered, and further characterization of these modified proteins will shed light on the significance of O-GlcNAcylation biology.

## 6 Hexosamine biosynthesis and related pathways in response to stresses

**Figure 3:**
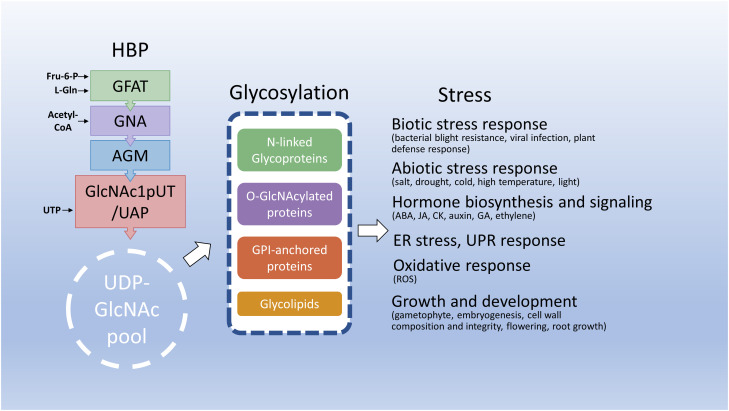
Hexosamine biosynthesis and related pathways in response to stresses. This diagram depicts that the HBP integrates several key metabolites to synthesize UDP-GlcNAc, an essential amino sugar moiety of glycosylation of proteins and lipids. Under stress conditions, HBP integrates endogenous metabolites and energy status to maintain UDP-GlcNAc homeostasis and reprogram metabolic pathways including glycosylation to benefit plant adaptation to deleterious environments. Fru-6-P, fructose-6-phosphate; L-Gln, L-glutamine; CoA, coenzyme A; UTP, uridine triphosphate; ABA, abscisic acid; JA, jasmonic acid; CK, cytokinin; GA, gibberellic acid.

UDP-GlcNAc biosynthesis through HBP is essential for the glycosylation of proteins and lipids (Ebert et al., 2018). Thus, the endogenous levels of UDP-GlcNAc levels intimately affect the glycosylation of proteins and lipids. For example, partial loss-of-function mutations in HBP-related genes normally reduce UDP-GlcNAc levels and impair N-glycosylation and/or O-GlcNAcylation of proteins (Jiang et al., 2005; Nozaki et al., 2012; Vu et al., 2019; Chen et al., 2022; Jia et al., 2023). Interestingly, these knockdown mutants largely display no apparent phenotype under normal growth conditions; however, these mutants exhibit stress-induced growth defects. This indicates that a small amount of UDP-GlcNAc is sufficient to maintain normal plant growth but more UDP-GlcNAc levels and protein glycosylation are needed for plants to adapt to deleterious environments. Most N-glycoproteins are membrane-associated and secreted proteins. Thus, changes in N-glycosylation through adverse environments or mutations of genes involved in HBP and N-glycan processing may alter glycoprotein functions in cell wall biosynthesis and integrity and membrane-associated proteins, resulting in altered sensitivities to biotic, such as bacterial blight tolerance (Wang et al., 2015), abiotic stresses, such as drought, salt, cold, and high temperature (Jiang et al., 2005; Xiao et al., 2018; Nozaki et al., 2012; Vu et al., 2019; Chen et al., 2022; Jia et al., 2023), or phytohormones, such as ABA, auxin, and JA (Zhang et al., 2009; Fanata et al., 2013; Wang et al., 2015; Jiao et al., 2020; Chen et al., 2022). Defects in N-glycoproteins might also cause the accumulation of unfolded or misfolded proteins in ER, leading to ER stress and further induction of UPR to enhance protein folding capacity and diminish ER stress (Bao and Howell, 2017; Yu et al., 2022). Therefore, ER stress or UPR can be observed in the mutations of HBP-related genes, such as *GFAT*, *GNA*, and *GlcNA.UT*s/or *UAP*/*SPL29* (Xiao et al., 2018; Nozaki et al., 2012; Vu et al., 2019; Chen et al., 2022) and N-glycan processing mutants, *stt3a* and *lew3* (Koiwa et al., 2003; Zhang et al., 2009). As UDP-GlcNAc is also essential for O-GlcNAcylation, defects in UDP-GlcNAc biosynthesis through HBP, such as AGM, or mutation of O-GlcNAcylation-related genes, such as *SEC*, might affect the functions of O-GlcNAcylated proteins, such as ATX1 and DELLA proteins, which further change temperature-dependent growth defects and cellular signalings, such as phytohormones ABA, GA, auxin, CK, and JA (Zentella et al., 2016; Jia et al., 2023), vernalization (Xiao et al., 2014; Xu et al., 2019), and viral infection (Pérez Jde et al., 2013; Martínez-Turiño et al., 2018). Hexosamine biosynthesis and related pathways in response to stresses are summarized in **Figure 3**.

## 7 Conclusions and future perspectives

Although the HBP is considered a minor side pathway of glycolysis, it integrates the endogenous nutrient status of plants and rewires the metabolic programs to improve plant development and adaption to environmental challenges. Thus, HBP serves as a metabolic integrator or sensor to fine-tune the nutrient balance and maintain UDP-GlcNAc homeostasis. Dysfunction of HBP often causes severe phenotypes or even lethality. Research progress on HBP in plants has been much slower than that in microbes and mammals. To date, despite HBP’s studies having made a great step in plants, several aspects remain to be further explored in the future.

1. HBP-related enzymatic proteins, such as GlcNAc1pUT2, UAP1/SPL29, and AtAGM, often have multiple substrates and products (or intermediates). The functions of these products’ targets or conjugates remain to be illustrated.
2. In addition to the cytosol and ER surface of the cytosolic side, enzymes, such as AGM and GlcNAc1pUT1, have several subcellular localizations, such as nuclei and organelles. It remains to be determined whether these proteins perform additional functions in addition to their involvement in the HBP.
3. How nutrient availability and environmental conditions control the HBP flux needs to be further examined in plants.
4. The total proteins of N-glycosylation and O-GlcNAcylation were underestimated in plants compared to mammals. Thus, high-throughput analysis of more GlcNAc-conjugated proteins needs to be performed, and the functions of these modified proteins remain to be characterized.
5. In addition to phosphorylation, O-GlcNAcylation sites of proteins can also compete with other post-translational modifications. The biological functions of these modified proteins also need to be unraveled in the future.

A better understanding of the functions of HBP, GlcNAc conjugates, and the mechanisms by which HBP responds to abiotic stress will reveal possible strategies to modify HBP in the biofortification of agriculture in the future.

## Author contributions

Y-HC: Data curation, Writing – original draft. W-HC: Conceptualization, Funding acquisition, Writing – review & editing.
